# Supplementary material for: Spatially controlled construction of assembloids using bioprinting
Source: Nat Commun. 2023 Jul 19;14:4346. doi: 10.1038/s41467-023-40006-5 (PMC10356773; doi:10.1038/s41467-023-40006-5)
Supplement: Supplementary file 1 — Supplementary Information [file 41467_2023_40006_MOESM1_ESM.pdf]

Supplementary Information for

Spatially controlled construction of assembloids using bioprinting

Julien G. Roth[1,2,3], Lucia G. Brunel[4,†], Michelle S. Huang[4,†], Yueming Liu[5], Betty Cai[5], Sauradeep Sinha[6], Fan Yang[6,7], Sergiu P. Pașca[2,8], Sungchul Shin[5], Sarah C. Heilshorn[2,5,*]

1 Institute for Stem Cell Biology and Regenerative Medicine, Stanford University School of Medicine, Stanford, CA, USA
2 Stanford Brain Organogenesis, Wu Tsai Neurosciences Institute & Bio-X, Stanford University, Stanford, CA, USA
3 Complex in Vitro Systems, Safety Assessment, Genentech Inc., South San Francisco, CA, USA
4 Department of Chemical Engineering, Stanford University, Stanford, CA, USA
5 Department of Materials Science and Engineering, Stanford University, Stanford, CA, USA
6 Department of Bioengineering, Stanford University, Stanford, CA, USA
7 Department of Orthopedic Surgery, Stanford University School of Medicine, Stanford, CA, USA
8 Department of Psychiatry and Behavioral Sciences, Stanford University, Stanford, CA, USA
† These authors contributed equally to this work
* Person to whom correspondence should be addressed, heilshorn@stanford.edu

This PDF file includes:

Supplementary Figure 1. Dorsal and ventral forebrain organoids express region-specific markers.
Supplementary Figure 2. Neural organoid apparent surface tension measurements.
Supplementary Figure 3. Neural organoid viability following vacuum aspiration and magnetic lifting.
Supplementary Figure 4. Aspiration-mediated deformation over time.
Supplementary Figure 5. Aspiration disrupts the internal cytoarchitecture of hiPSC-derived neural organoids.
Supplementary Figure 6. CNF MNPs coat neural organoids sufficiently for lifting.
Supplementary Figure 7. Quantification of magnetic field strength.
Supplementary Figure 8. Orbital shakers coat neural organoids sufficiently for lifting.
Supplementary Figure 9. The effect of MNPs on neural organoids is limited.
Supplementary Figure 10. Modulus recovery following high strain for CNF support scaffolds.
Supplementary Figure 11. Cellulase degrades CNF in a bioorthogonal manner.
Supplementary Figure 12. Cellulase-mediated removal of residual CNF.
Supplementary Figure 13. MNP surface coverage throughout SPOT.
Supplementary Figure 14. SPOT chip design and fabrication.
Supplementary Figure 15. XYZ localization with SPOT.
Supplementary Figure 16. Three-part fusion of two regionalized neural organoids and a DIPG organoid.
Supplementary Figure 17. Infiltration of a DIPG organoid into a neural organoid.
Supplementary Figure 18. Neural and glial cell types surrounding DIPG organoid infiltration into neural organoids.
Supplementary Figure 19. DIPG organoids fused to an array of neural organoids.
Supplementary Figure 20. Panobinostat does not induce substantial apoptosis in neural organoids.
Supplementary Figure 21. A collection of brain tumor organoids fused to a neural organoid.
Supplementary Method 1 Ink Extrusion G-Code
Supplementary Method 2 Control of Magnetic Rod and Electromagnetic Field
Supplementary Table 1. Previous demonstrations of AAB.

Supplementary Table 2. Characterization of MNPs.
Supplementary Table 3. Fluorescence recovery after photobleaching of 0.5 wt% CNF across MWs.
Supplementary Table 4. Primer sequences.

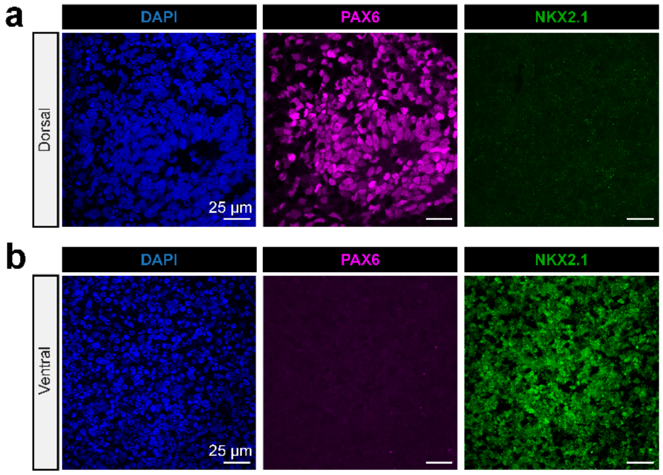

Supplementary Figure 1. Dorsal and ventral forebrain organoids express region-specific markers.
a. Representative IF images of a dorsal forebrain neural organoid stained for dorsal progenitor cell (PAX6) and ventral (NKX2.1) fate.
b. Representative IF images of a ventral forebrain neural organoid stained for PAX6 and NKX2.1.

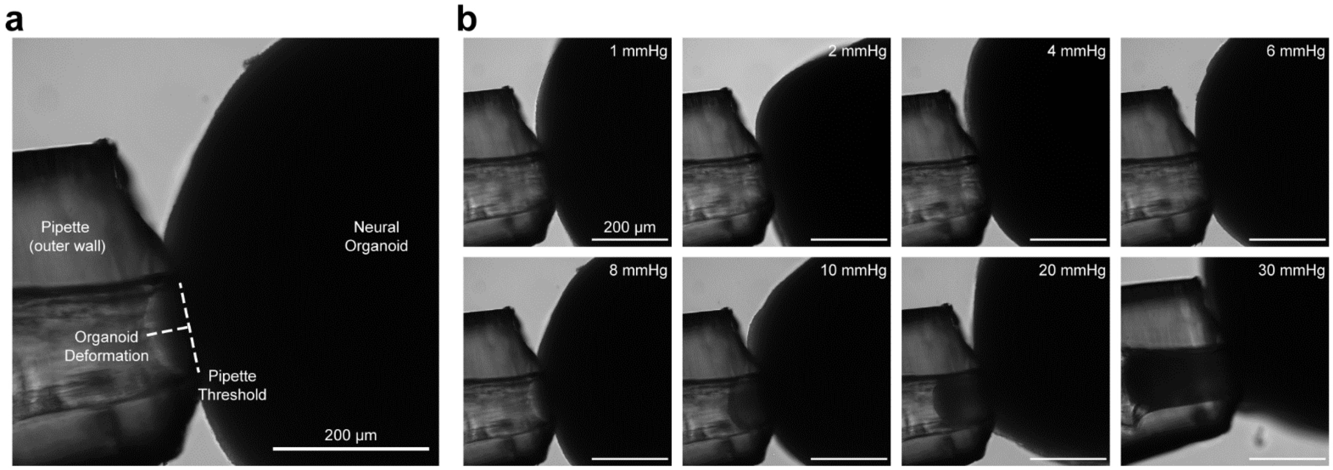

Supplementary Figure 2. Neural organoid apparent surface tension measurements.
a. Representative BF image of a neural organoid adjacent to the vacuum aspirator used for apparent surface tension measurements.
b. Representative BF images of the same neural organoid across a range of vacuum pressures.

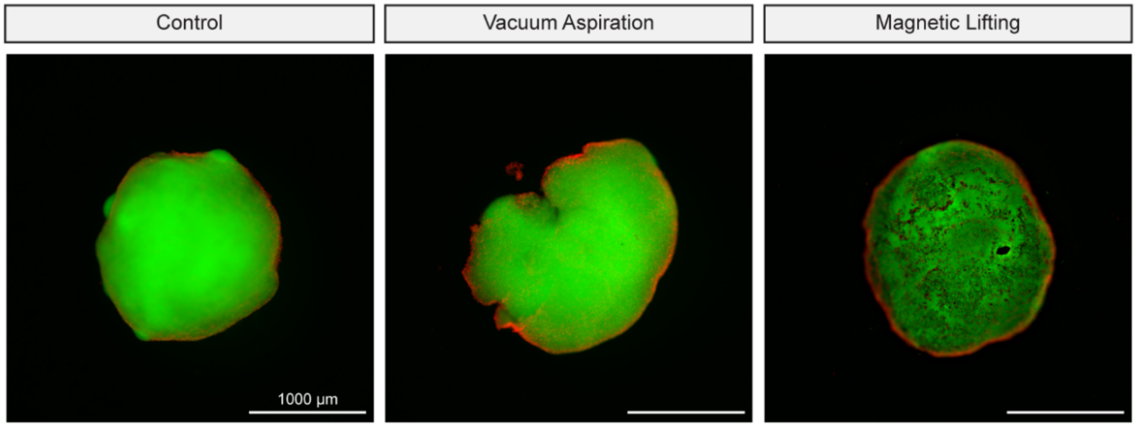

Supplementary Figure 3. Neural organoid viability following vacuum aspiration and magnetic lifting.

Representative fluorescence images of neural organoids with and without aspiration-assisted lifting and magnetic lifting with calcein-AM labeled live cells (green) and ethidium homodimer-1 labeled dead cells (red). The control organoid was not lifted. The dark dots visible on the neural organoid exposed to magnetic lifting are MNPs on the organoid surface.

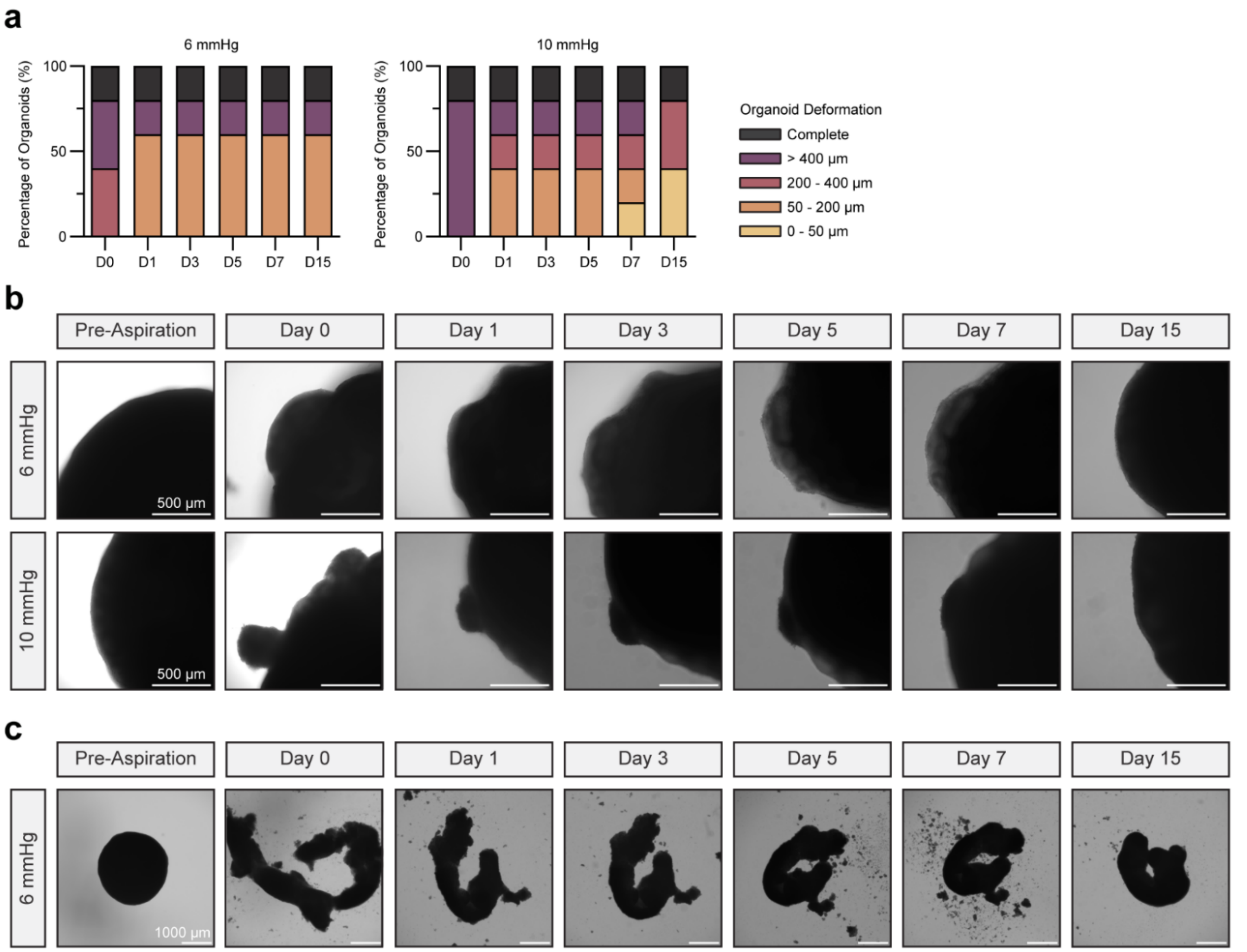

Supplementary Figure 4. Aspiration-mediated deformation over time.

a. Quantification of neural organoid deformation over time following exposure to either 6 mmHg or 10 mmHg vacuum pressure (n = 5 per vacuum pressure).

b. Representative BF images of a single neural organoid exposed to either 6 mmHg or 10 mmHg over time.

c. Representative BF images of a single neural organoid which, when exposed to 6 mmHg, exhibited a deformation so severe that it was no longer spherical, herein referred to as ‘Complete Deformation’.

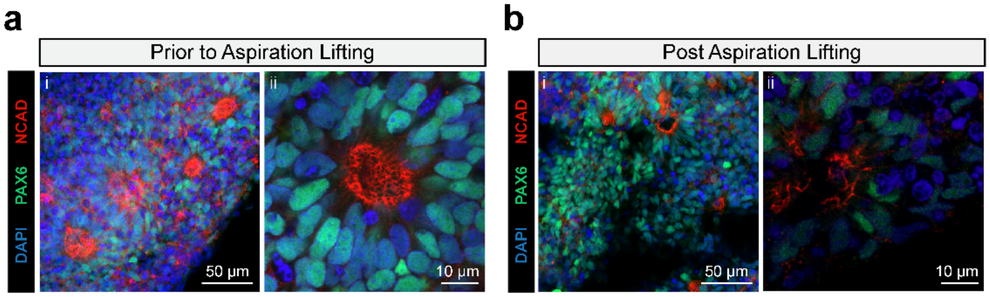

Supplementary Figure 5. Aspiration disrupts the internal cytoarchitecture of hiPSC-derived neural organoids.

a. Representative IF images of a neural organoid prior to vacuum aspiration where PAX6-expressing dorsal progenitor cells and lumens lined by cells expressing the apical adherens junction marker NCAD.

b. Representative IF images of a neural organoid post vacuum aspiration (6 mmHg).

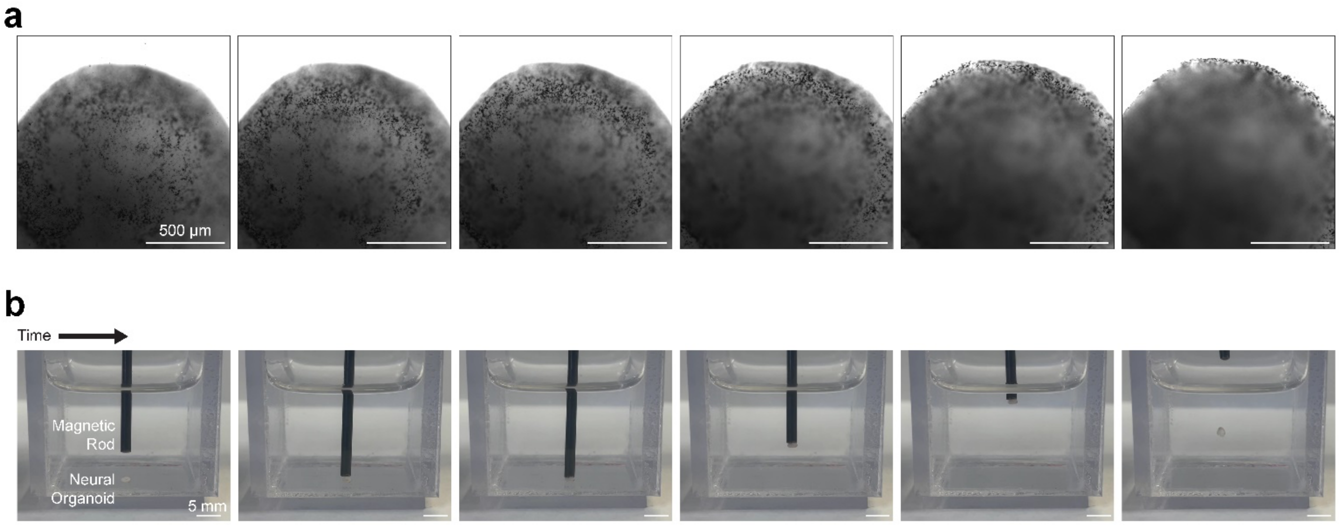

Supplementary Figure 6. CNF MNPs coat neural organoids sufficiently for lifting.
a. Representative BF images of different z-planes of a single neural organoid coated with an MNP-laden CNF ink.
b. Representative images of the same neural organoid being lifted and released by an iron rod affixed to an electromagnet.

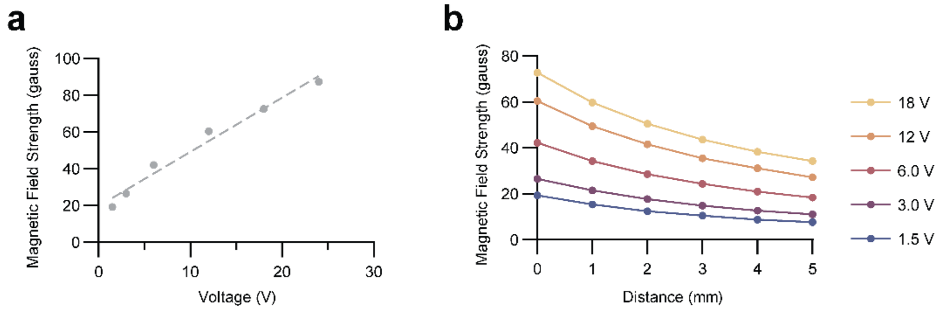

Supplementary Figure 7. Quantification of magnetic field strength.

a. Magnetic field strength over increasing voltages. Line of best fit: $y = 2.95x + 19.67$. Coefficient of determination: $r^2 = 0.98$.

b. Magnetic field strength at distinct voltages over increasing distances.

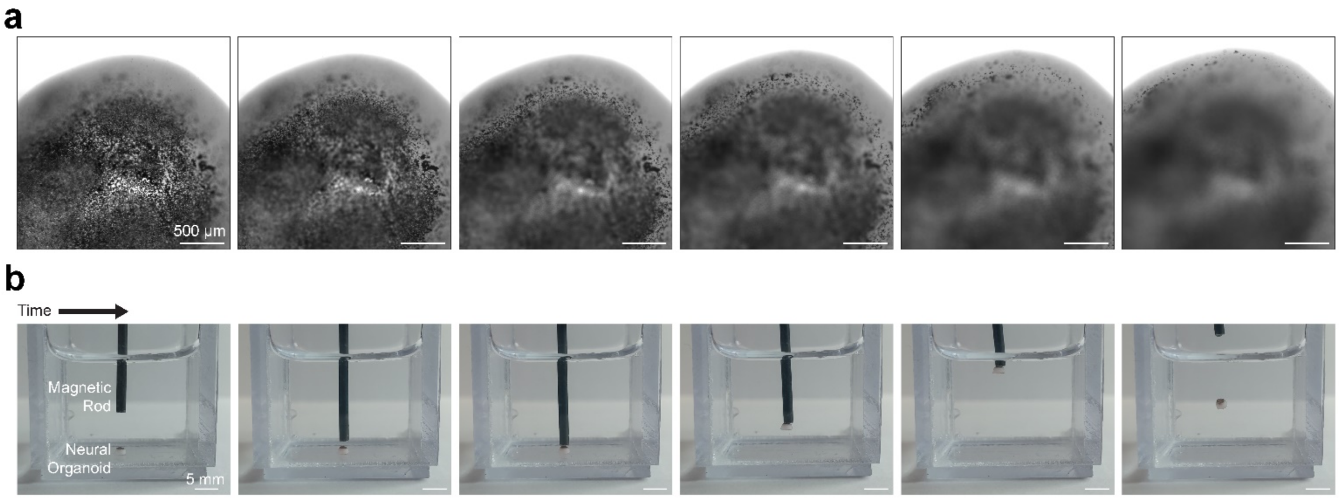

Supplementary Figure 8. Orbital shakers coat neural organoids sufficiently for lifting.
a. Representative BF images of different z-planes of a single neural organoid incubated in an orbital shaker with MNPs in DPBS.
b. Representative images of the same neural organoid being lifted and released by an iron rod affixed to an electromagnet.

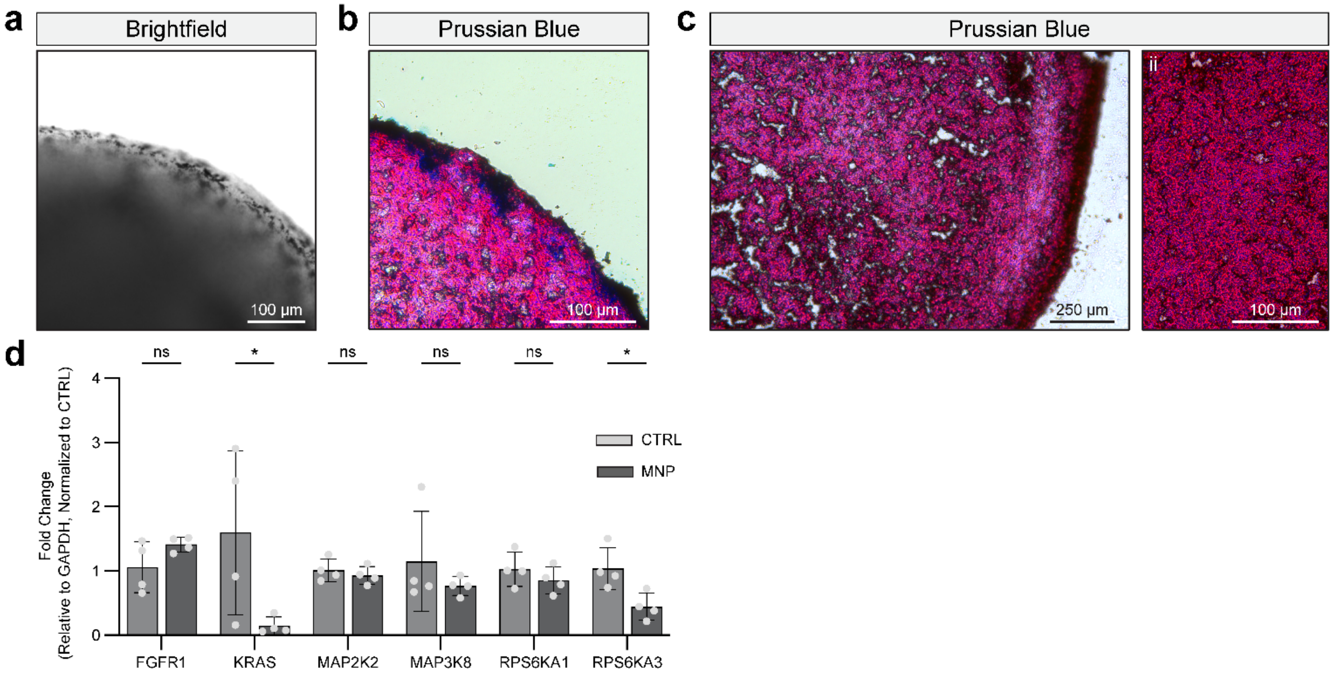

Supplementary Figure 9. The effect of MNPs on neural organoids is limited.

a. Representative BF images of a single z-plane of a neural organoid coated with MNPs.

b. Representative Prussian blue staining of an MNP-coated neural organoid wherein blue denotes iron.

c. Representative Prussian blue staining of a neural organoid that has never come in contact with MNPs.

d. mRNA expression of MAPK genes previously identified to have been affected by iron oxide nanoparticles. Each data point represents a different hiPSC-derived neural organoid ($n = 4$). CTRL represent non-coated organoids while MNP represent MNP-coated organoids. p values for each transcript comparison are as follows: FGFR1 $p = 0.1777$, KRAS $p = 0.0375$, MAP2K2 $p = 0.489$, MAP3K8 $p = 0.3999$, RPS6KA1 $p = 0.3506$, RPS6KA3 $p = 0.0264$

Statistical analyses performed separately for each marker with unpaired, two-tailed t tests with Welch's correction. Data plotted as mean ± SD where * $p<0.05$, ** $p<0.01$, *** $p<0.001$, **** $p<0.0001$, and ns = not significant.

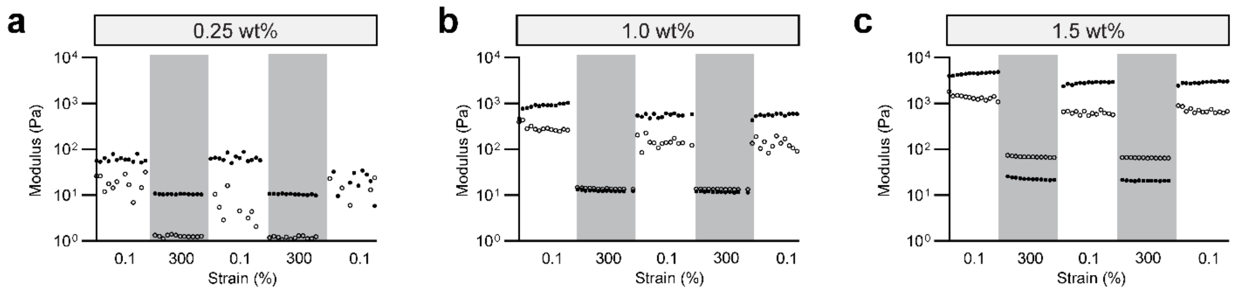

Supplementary Figure 10. Modulus recovery following high strain for CNF support scaffolds.
a. Representative storage modulus (filled circles) and loss modulus (open circles) of 0.25 wt% CNF support scaffold exposed to cyclical periods of low (0.1 %) and high (300 %) strain to evaluate the ability of the material to shear thin and self-heal. Percent G' recovery following one cycle (mean ± SD): 108.6 ± 66.2 (n = 4 gels); percent G' recovery following two cycles (mean ± SD): 67.6 ± 27.7 (n = 4 gels).
b. Representative storage modulus and loss modulus of 1.0 wt% CNF support scaffold exposed to cyclical periods of low and high strain. Percent G' recovery following one cycle (mean ± SD): 66.3 ± 8.4 (n = 4 gels); percent G' recovery following two cycles (mean ± SD): 63.7 ± 10.3 (n = 4 gels).
c. Representative storage modulus and loss modulus of 1.5 wt% CNF support scaffold exposed to cyclical periods of low and high strain. Percent G' recovery following one cycle (mean ± SD): 61.9 ± 1.2 (n = 4 gels); percent G' recovery following two cycles (mean ± SD): 60.4 ± 3.7 (n = 4 gels).

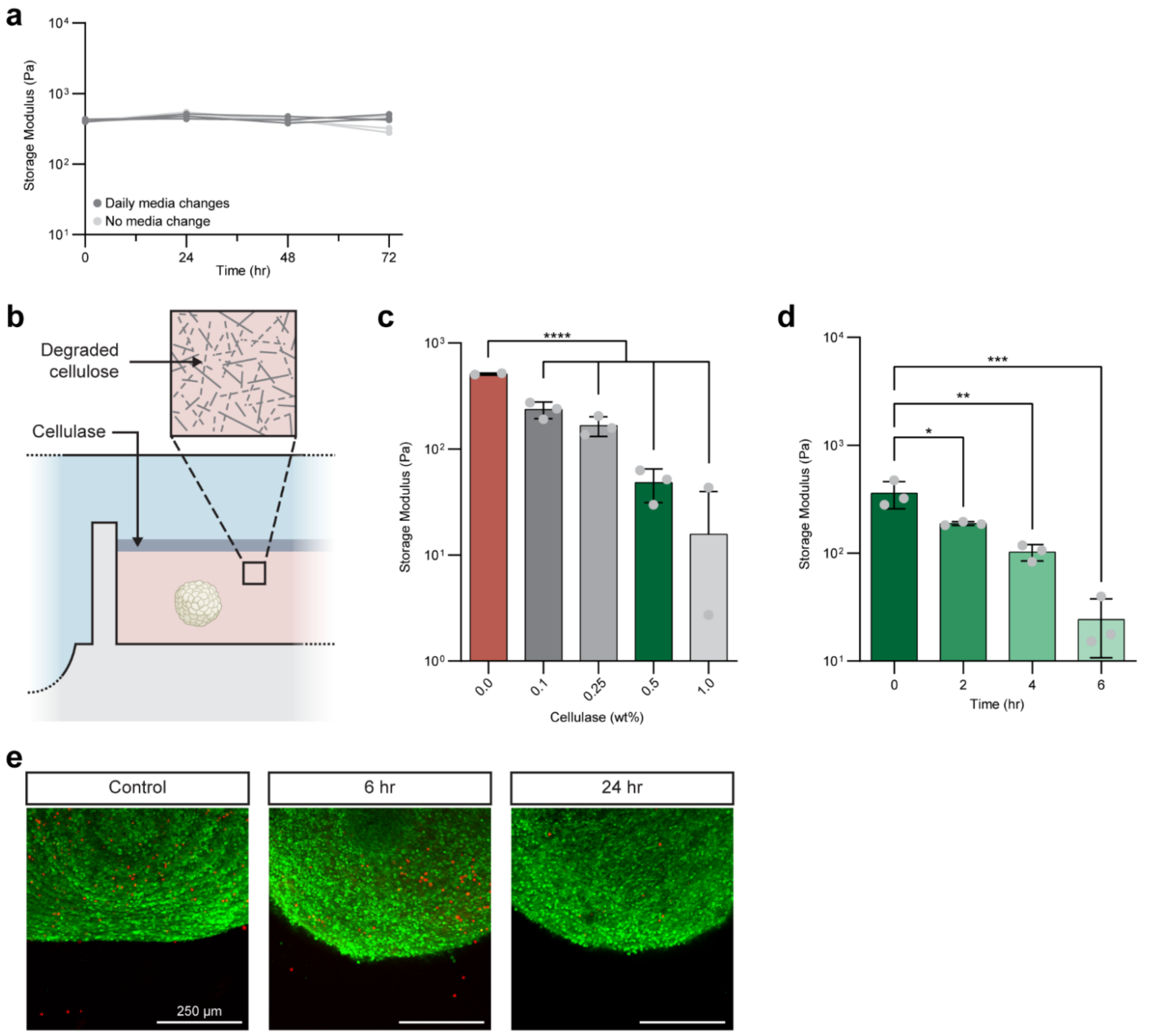

Supplementary Figure 11. Cellulase degrades CNF in a bioorthogonal manner.

a. Storage modulus of 0.5 wt% CNF support scaffold over time with or without daily media changes.

b. Schematic of the use of cellulase to degrade CNF.

c. Storage modulus of 0.5 wt% CNF exposed to various wt% of cellulase (n = 3 hydrogels). p values for each comparison are as follows: Control vs. 1mg/ml $p < 0.0001$, Control vs. 2.5mg/ml $p < 0.0001$, Control vs. 5mg/ml $p < 0.0001$, Control vs. 10mg/ml $p < 0.0001$

d. Storage modulus of 0.5 wt% CNF exposed to 0.5 wt% cellulase over time (n = 3 hydrogels). p values for each comparison are as follows: 0 hr vs. 2 hr $p = 0.016$, 0 hr vs. 4 hr $p = 0.0014$, 0 hr vs. 6 hr $p = 0.0002$

e. Representative fluorescence images of neural organoids following 0.5 wt% cellulase treatment over time with calcein-AM labeled live cells (green) and ethidium homodimer-1-labeled dead cells (red).

Statistical analyses performed as one-way ANOVA with Tukey multiple comparisons test. Data plotted as mean ± SD where * $p<0.05$, ** $p<0.01$, *** $p<0.001$, **** $p<0.0001$, and ns = not significant.

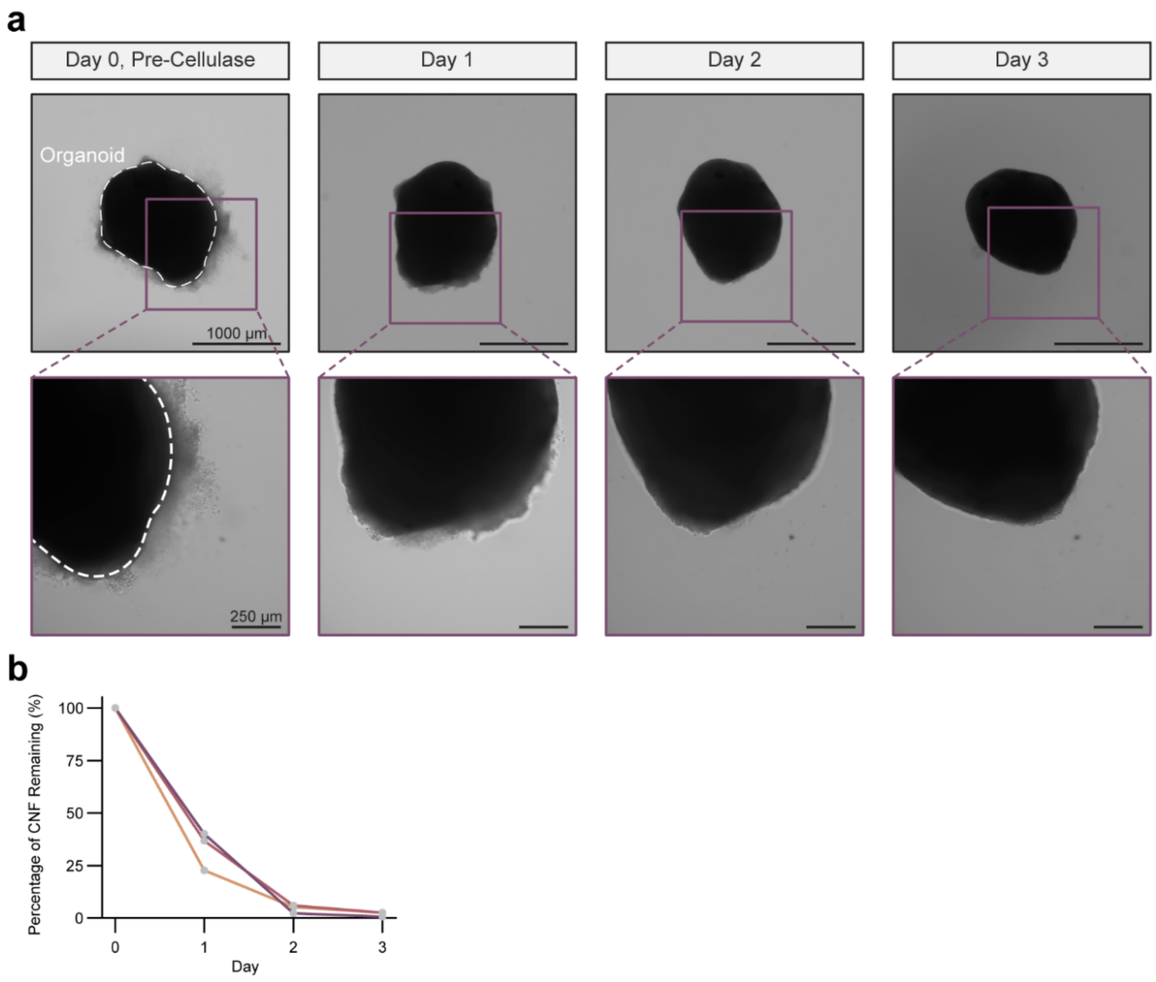

Supplementary Figure 12. Cellulase-mediated removal of residual CNF.
a. Representative BF images of a neural organoid following SPOT and removal from the CNF support scaffold. The organoid was treated with 0.5 wt% cellulase over 3 days to remove residual CNF on the organoid's surface.
b. Quantification of CNF removal with 0.5 wt% cellulase. Each set of data points connected with a line represents a single replicate.

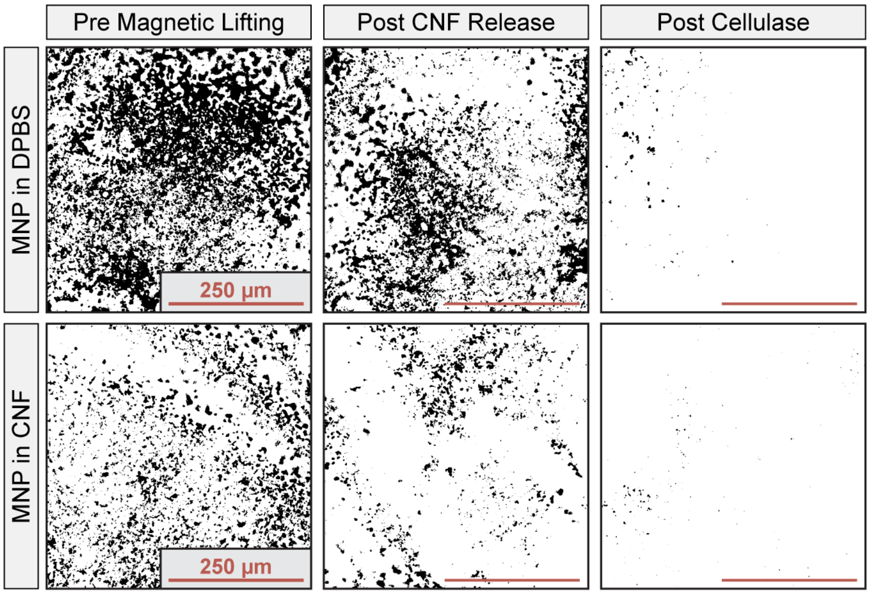

Supplementary Figure 13. MNP surface coverage throughout SPOT.
Representative maximum projection, false colored BF images of neural organoid surfaces following SPOT wherein one organoid was coated with MNPs suspended in DPBS and the other with an MNP-laden 0.025 wt% CNF ink. In both cases, the initial concentration of MNPs in the coatings were 1 wt%.

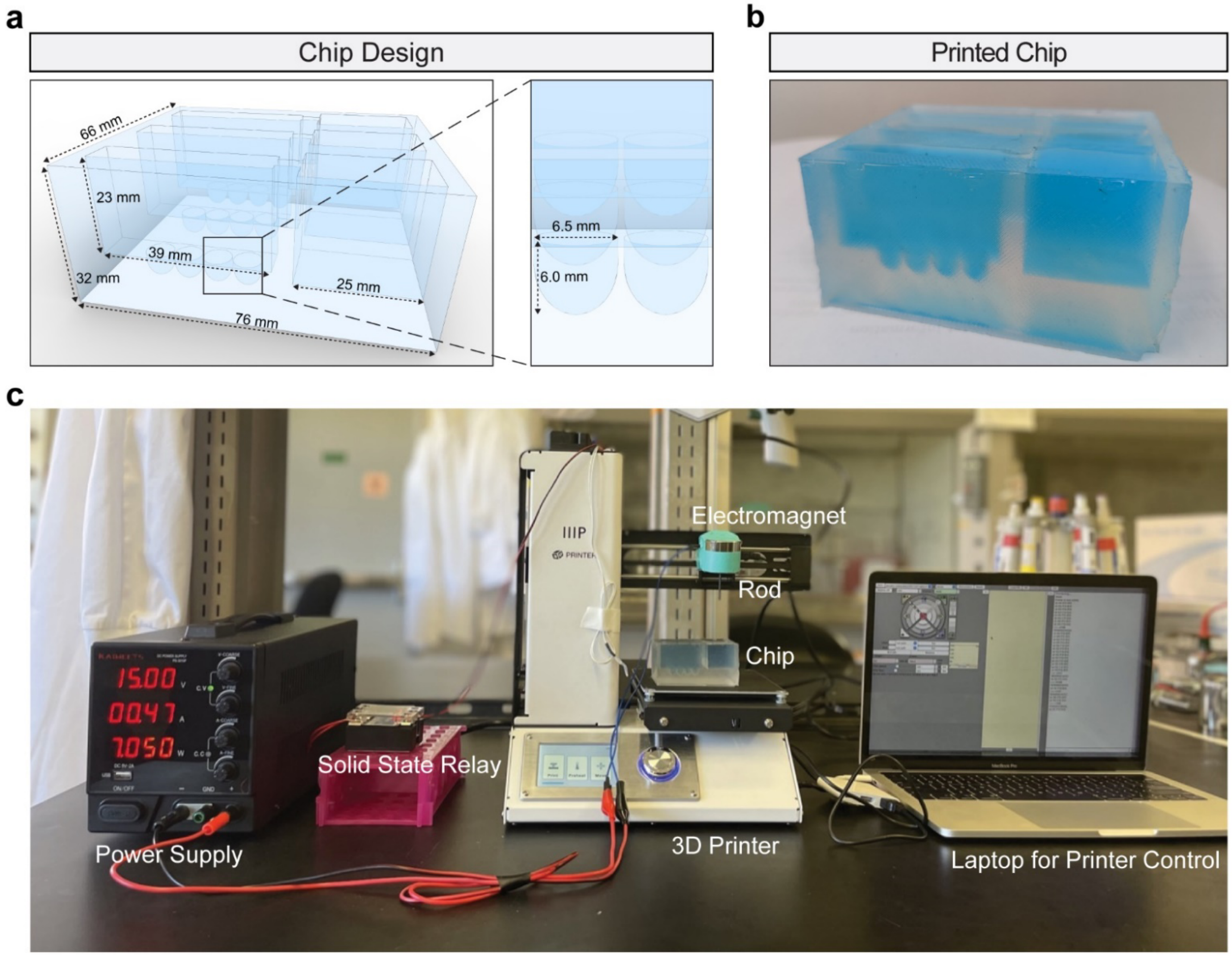

Supplementary Figure 14. SPOT chip design and fabrication.
a. 3D rendering with measurements of the chip designed to facilitate SPOT automation.
b. Image of the chip after the PDMS has been cured. The reservoirs are filled with DPBS with blue food coloring to provide contrast.
c. Image of the SPOT set-up.

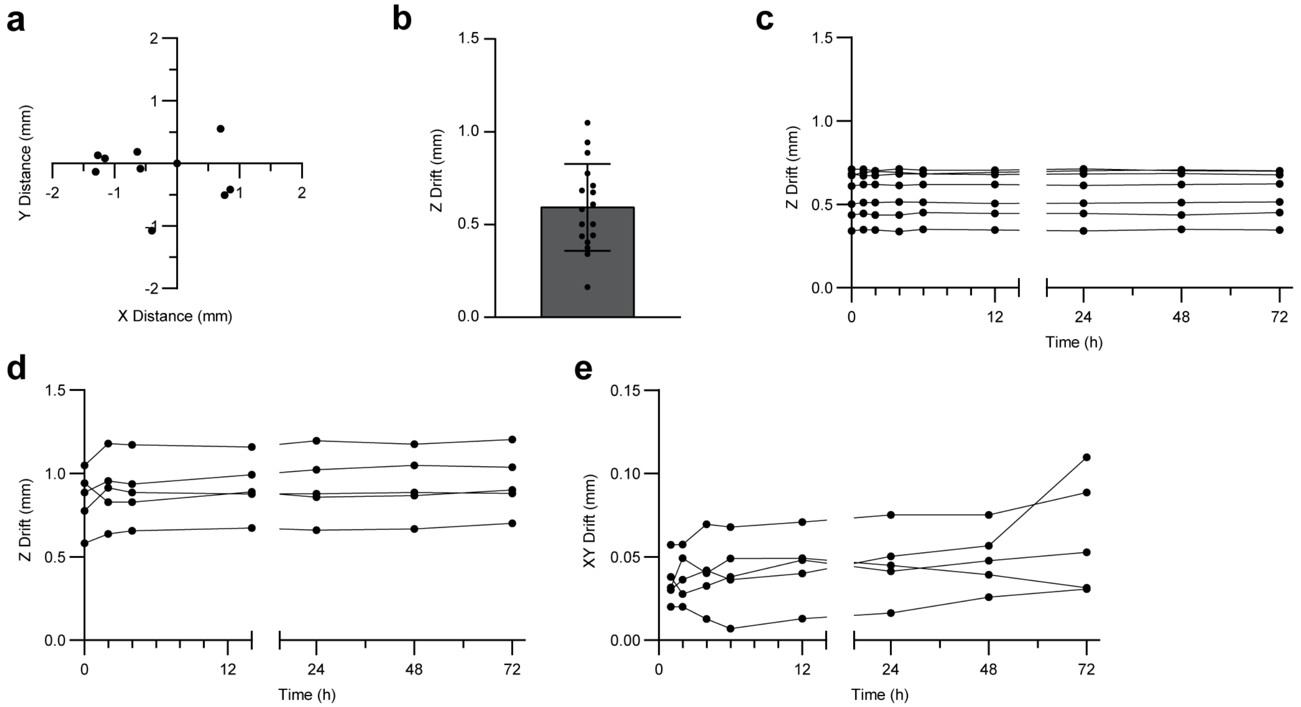

Supplementary Figure 15. XYZ localization with SPOT.
a. Precision of automated SPOT positioning in X and Y dimensions. Data points represent the observed positions where alginate microgels were deposited in a coordinate plane wherein (0,0) represents the intended position of the microgel.
b. Quantification of the initial Z drift. Each data point represents a different organoid (n = 17) and the data are plotted as mean ± SD.
c. Z drift of microgels with diameters greater than 1.5 mm over time.
d. Z drift of microgels with diameters less than 1.5 mm over time.
e. Total XY drift of microgels over time.

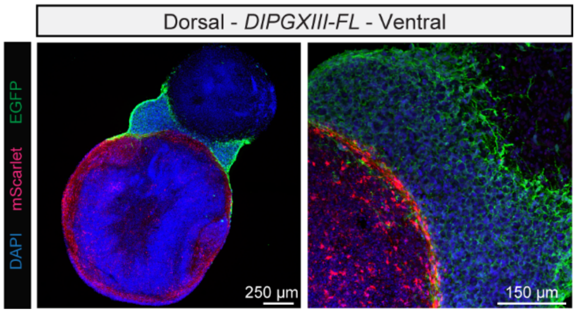

Supplementary Figure 16. Three-part fusion of two regionalized neural organoids and a DIPG organoid.

Representative IF images of an mScarlet-expressing dorsal forebrain neural organoid fused to an eGFP-expressing frontal lobe DIPG metastasis fused to a non-fluorescent ventral forebrain neural organoid one-week post-fusion.

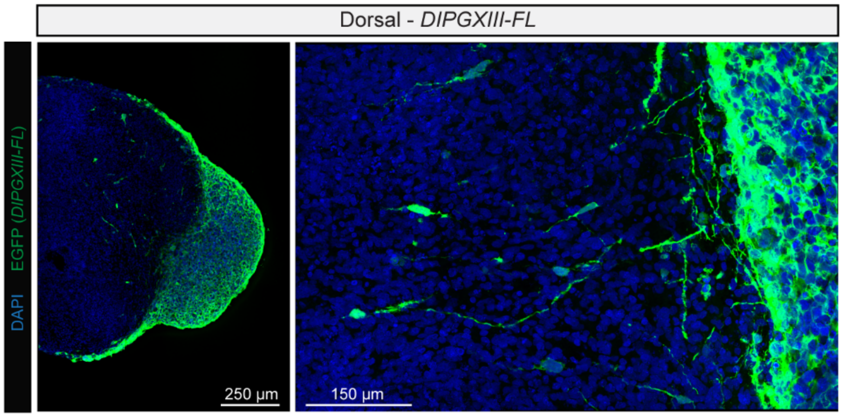

Supplementary Figure 17. Infiltration of a DIPG organoid into a neural organoid.
Representative IF images of an eGFP-expressing frontal lobe DIPG metastasis fused to a non-fluorescent dorsal forebrain neural organoid one-week post-fusion.

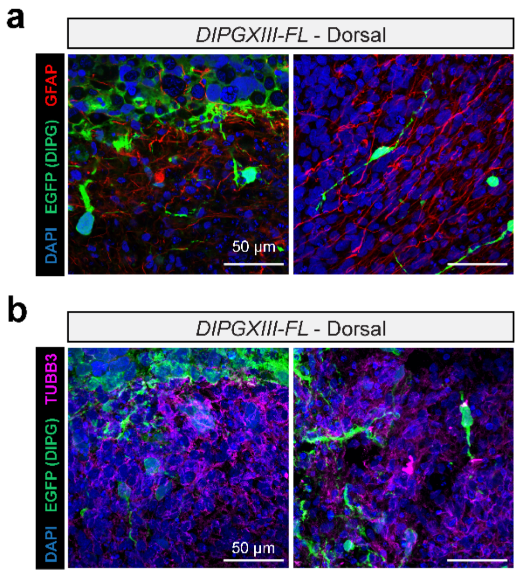

Supplementary Figure 18. Neural and glial cell types surrounding DIPG organoid infiltration into neural organoids.

a. Representative IF images of an eGFP-expressing frontal lobe DIPG metastasis infiltrating a non-fluorescent dorsal forebrain neural organoid with staining for glial cells (GFAP).

b. Representative IF images of an eGFP-expressing frontal lobe DIPG metastasis infiltrating a non-fluorescent dorsal forebrain neural organoid with staining for neural projections (TUBB3).

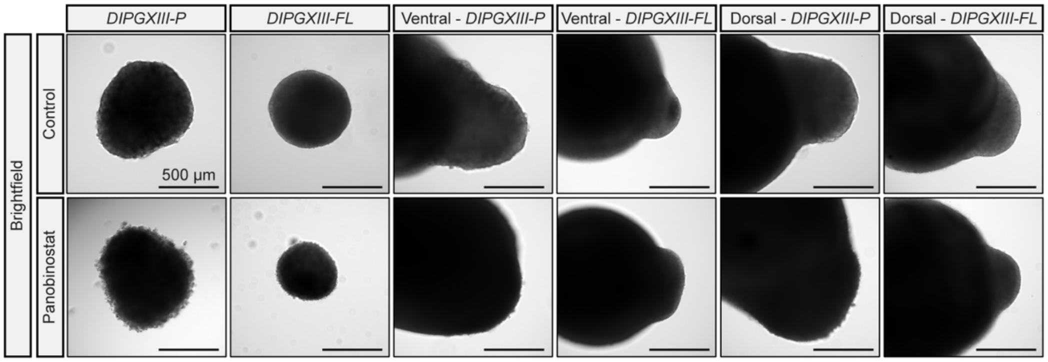

Supplementary Figure 19. DIPG organoids fused to an array of neural organoids.
Representative BF images of DIPG organoids, from the pons and frontal lobe, fused to ventral and dorsal neural organoids with and without 200 nM panobinostat.

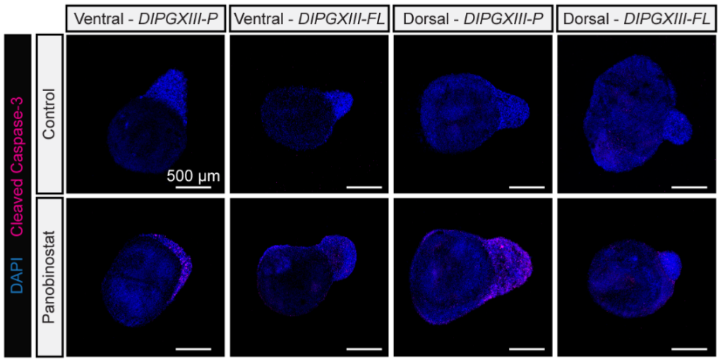

Supplementary Figure 20. Panobinostat does not induce substantial apoptosis in neural organoids.

Representative IF images stained with the apoptosis marker cleaved caspase-3 of DIPG organoids, from the pons and frontal lobe, fused to ventral and dorsal neural organoids with and without 200 nM panobinostat.

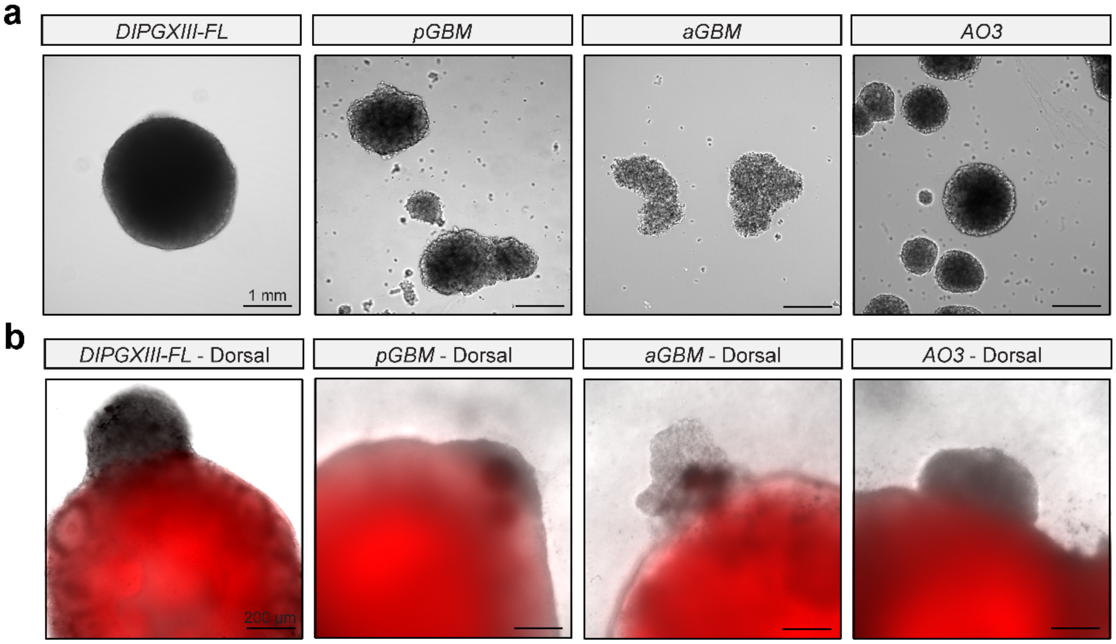

Supplementary Figure 21. A collection of brain tumor organoids fused to a neural organoid.
a. Representative BF images of DIPG, pediatric glioblastoma (pGBM), adult glioblastoma (aGBM), and anaplastic oligodendroglioma (AO3) organoids.
b. Representative BF images of mScarlet-expressing dorsal forebrain neural organoids fused to primary brain tumor organoids one day post-fusion.

Supplementary Method 1. Ink Extrusion G-Code

```
;start gcode
M83
G92 X0 Y0 Z0 E0

G1 F25 X0 Y0 Z0 E0.25 ; deposits ink on first organoid
G1 F25 X0 Y0 Z-0.5
G1 F25 X0 Y0 Z0
G1 F25 X0 Y0 Z-0.5
G1 F25 X0 Y0 Z0
G1 F300 X0 Y0 Z8 ; lifts print head and moves to second organoid
G1 X7 Y0 Z8
G1 X7 Y0 Z0
G1 F25 X7 Y0 Z-0.5
G1 X7 Y0 Z0 E0.25 ; deposits ink on second organoid
G1 X7 Y0 Z-0.5
G1 F300 X7 Y0 Z0
G1 X7 Y0 Z8 ; lifts print head and moves to third organoid
G1 X14 Y0 Z8
G1 X14 Y0 Z0
G1 F25 X14 Y0 Z-0.5
G1 X14 Y0 Z0 E0.25 ; deposits ink on third organoid
G1 X14 Y0 Z-0.5
G1 F300 X14 Y0 Z0
G1 X14 Y0 Z0
G1 X14 Y0 Z8 ; lifts print head and moves to fourth organoid
G1 X21 Y0 Z8
G1 X21 Y0 Z0
G1 F25 X21 Y0 Z-0.5
G1 X21 Y0 Z0 E0.25 ; deposits ink on fourth organoid
G1 F300 X21 Y0 Z-0.5
G1 X21 Y0 Z0
G1 X21 Y0 Z8

G1 F1200 X10 Y0 Z20
G1 F1200 X0 Y0 Z20 ; raises print head above initial position
```

Supplementary Method 2. Control of Magnetic Rod and Electromagnetic Field

```
;start gcode
M83
G92 X0 Y0 Z0 E0

M106 ; turns on electromagnet
G1 F600 X0 Y0 Z0 ; lifts first organoid
G1 X0 Y0 Z-0.5
G1 F600 X0 Y0 Z0
G1 X0 Y0 Z-0.5
G1 F2000 X0 Y0 Z17 ; transfers first organoid
G1 X40.5 Y0 Z17
G1 F600 X40.5 Y0 Z8
M107 ; turns off electromagnet
G1 X40.5 Y0 Z7.5 ; deposits first organoid
G1 X40.5 Y0 Z8
G1 X40.5 Y0 Z7.5
G1 F2000 X40.5 Y0 Z7.5
G1 X40.5 Y0 Z17

M106 ; turns on electromagnet
G1 X7 Y0 Z17
G1 F600 X7 Y0 Z0 ; lifts second organoid
G1 X7 Y0 Z-0.5
G1 F600 X7 Y0 Z0
G1 X7 Y0 Z-0.5
G1 F2000 X7 Y0 Z17 ; transfers second organoid
G1 X41.5 Y0 Z17
G1 F600 X41.5 Y0 Z8
M107 ; turns off electromagnet
G1 X41.5 Y0 Z7.5 ; deposits second organoid
G1 X41.5 Y0 Z8
G1 X41.5 Y0 Z7.5
G1 F2000 X41.5 Y0 Z7.5
G1 X41.5 Y0 Z17

M106 ; turns on electromagnet
G1 F600 X14 Y0 Z17
G1 X14 Y0 Z0
G1 F600 X14 Y0 Z0 ; lifts third organoid
G1 X14 Y0 Z-0.5
G1 F600 X14 Y0 Z0
G1 X14 Y0 Z-0.5
G1 F2000 X14 Y0 Z17 ; transfers third organoid
G1 X42.5 Y0 Z17
G1 F600 X42.5 Y0 Z8
```

```
M107 ; turns off electromagnet
G1 X42.5 Y0 Z7.5 ; deposits third organoid
G1 X42.5 Y0 Z8
G1 X42.5 Y0 Z7.5
G1 F2000 X42.5 Y0 Z7.5
G1 X42.5 Y0 Z17
M106 ; turns on electromagnet
G1 X21 Y0 Z17
G1 X21 Y0 Z0
G1 F600 X21 Y0 Z0 ; lifts fourth organoid
G1 X21 Y0 Z-0.5
G1 F600 X21 Y0 Z0
G1 X21 Y0 Z-0.5
G1 F2000 X21 Y0 Z17 ; transfers fourth organoid
G1 X43.5 Y0 Z17
G1 F600 X43.5 Y0 Z8
M107 ; turns off electromagnet
G1 X43.5 Y0 Z7.5 ; deposits fourth organoid
G1 X43.5 Y0 Z8
G1 X43.5 Y0 Z7.5
G1 F2000 X43.5 Y0 Z7.5
G1 X43.5 Y0 Z17

G1 F2000 X10 Y0 Z20
G1 F2000 X0 Y0 Z20 ; raises print head above initial position
```

Supplementary Table 1. Previous demonstrations of AAB.

Cell Type	Spheroid Size (µm)	Vacuum Pressure (mmHg)	Nozzle Diameter (µm)	Support Type	Dimensionality	Reference
MSC, HUVEC, 3T3, 4T1, HDF	200-600	20-60	250	Agarose	2D	Ayan, Ozbolat et al. *Sci. Adv.* 2020.
MSC	150-400	20-300	150	Carbopol	3D	Ayan, Ozbolat et al. *Commun. Phys.* 2020.
MSC	250-600	70	200	Alginate	3D	Kim, Ozbolat et al. *Biofabrication.* 2022.
MSC, hiPSC-CM	200-400	Not described	100	Hyaluronan	3D	Daly, Burdick et al. *Nat. Commun.* 2021.

Aspiration-assisted bioprinting (AAB)
Mesenchymal stromal cells (MSC),
Human umbilical vein endothelial cells (HUVEC),
NIH/3T3 murine embryonic fibroblasts (3T3),
Murine breast cancer cell line derived from mammary gland (4T1),
Human dermal fibroblasts (HDF),
Human induced pluripotent stem cell-derived cardiomyocytes (hiPSC-CM)

Supplementary Table 2. Characterization of MNPs.

	Homemade (mean ± SD)	Commercial (mean ± SD)
Zeta Potential (mV)	-22.26 ± 1.342	49.50 ± 5.920 *
Aggregate Diameter (nm)	2864 ± 4438	2545 ± 1926
Hydrodynamic Diameter (nm)	1003 ± 49.3	669.8 ± 41.0

Magnetic nanoparticles (MNPs)
* These values provided by Alpha Nanotech

Supplementary Table 3. Fluorescence recovery after photobleaching of 0.5 wt% CNF across MWs.

Diffusant MW (Da)	Hydrodynamic Radius (nm)	Diffusivity (um^2 s^-1)
10,000	2.3	130 ± 9
20,000	3.3	105 ± 2
40,000	4.5	82 ± 2
70,000	6.0	85 ± 3
150,000	8.5	82 ± 2
250,000	10.6	63 ± 2
500,000	14.7	39 ± 1

Percent by weight (wt%)
Cellulose nanofiber (CNF)
Molecular weight (MW)

Supplementary Table 4. Primer sequences.

Target	Forward Primer (5' to 3')	Reverse Primer (5' to 3')
FGFR1	CGTGGCCTTGACCTCCAACC	GTCCGCCATTGGCAAGCTGG
GAPDH	CATGAGAAGTATGACAACAGCCT	AGTCCTTCCACGATACCAAAGT
KRAS	GTCATGAACTGTACTACTCC	GGATACTTCTAACAAGCTGC
MAP2K2	GTCACGGGATGGATAGCCGG	CTCGGACCGCTTGATGAAGG
MAP3K8	CCTCACGACCACCTCATGAG	CACATGGTCATTAGACTGGG
RPS6KA1	CCACCAGGACCTACAGCTTG	CCTGTGGCCCGAATGCCCTG
RPS6KA3	CTTCAAGGTATTGCCATTCC	CCCCATTCCCACCCATGACC
