# Supplementary material for: Spatially controlled construction of assembloids using bioprinting
Source: Nat Commun. 2023 Jul 19;14:4346. doi: 10.1038/s41467-023-40006-5 (PMC10356773; doi:10.1038/s41467-023-40006-5)
Supplement: Supplementary file 3 — Description of Additional Supplementary Files [file 41467_2023_40006_MOESM3_ESM.pdf]

Description of Additional Supplementary Files

File Name: Supplementary Movie 1
Description: **Automated magnetic ink extrusion.** MNP-laden CNF hydrogel being disbursed via G-Code within a custom-designed SPOT chip.

File Name: Supplementary Movie 2
Description: **Automated control over magnetic rod movement and electromagnetic field.** G-code mediated movement of a magnetic rod and the electromagnet control unit. Movie has been set to 2X speed.

File Name: Supplementary Movie 3
Description: **Ventral interneuron migration into the dorsal forebrain organoid.** Representative IF of a section of a hiPSC-derived forebrain assembloid, constructed with SPOT, comprised of eGFP-expressing ventral and mScarlet-expressing dorsal neural organoids. DAPI (blue), mScarlet (magenta), eGFP (white).

File Name: Supplementary Movie 4
Description: **Interneurons exhibit highly branched morphologies within the dorsal forebrain organoid.** Representative IF of a section of a hiPSC-derived forebrain assembloid, constructed with SPOT, comprised of eGFP-expressing ventral and mScarlet-expressing dorsal neural organoids. DAPI (blue), mScarlet (magenta), eGFP (white).

File Name: Supplementary Movie 5
Description: **Interneurons span Z-depths within the dorsal forebrain organoid.** Representative IF with false-coloring to denote depth in the Z dimension of a section of a hiPSC-derived forebrain assembloid, constructed with SPOT, comprised of eGFP-expressing ventral and dorsal neural organoids. DAPI (blue), eGFP (white).

File Name: Supplementary Data 1
Description: **Statistical details.** Full statistical details for each figure panel.
