# Supplementary material for: Spatially controlled construction of assembloids using bioprinting
Source: Nat Commun. 2023 Jul 19;14:4346. doi: 10.1038/s41467-023-40006-5 (PMC10356773; doi:10.1038/s41467-023-40006-5)
Supplement: Supplementary file 10 — Reporting Summary [file 41467_2023_40006_MOESM10_ESM.pdf]

nature portfolio

Corresponding author(s): Sarah Christine Heilshorn

Last updated by author(s): 06.17.2023

Reporting Summary

Nature Portfolio wishes to improve the reproducibility of the work that we publish. This form provides structure for consistency and transparency in reporting. For further information on Nature Portfolio policies, see our Editorial Policies and the Editorial Policy Checklist.

Statistics

For all statistical analyses, confirm that the following items are present in the figure legend, table legend, main text, or Methods section.

n/a	Confirmed	
☐	☒	The exact sample size (*n*) for each experimental group/condition, given as a discrete number and unit of measurement
☐	☒	A statement on whether measurements were taken from distinct samples or whether the same sample was measured repeatedly
☐	☒	The statistical test(s) used AND whether they are one- or two-sided<br>*Only common tests should be described solely by name; describe more complex techniques in the Methods section.*
☒	☐	A description of all covariates tested
☐	☒	A description of any assumptions or corrections, such as tests of normality and adjustment for multiple comparisons
☐	☒	A full description of the statistical parameters including central tendency (e.g. means) or other basic estimates (e.g. regression coefficient) AND variation (e.g. standard deviation) or associated estimates of uncertainty (e.g. confidence intervals)
☐	☒	For null hypothesis testing, the test statistic (e.g. *F*, *t*, *r*) with confidence intervals, effect sizes, degrees of freedom and *P* value noted<br>*Give P values as exact values whenever suitable.*
☒	☐	For Bayesian analysis, information on the choice of priors and Markov chain Monte Carlo settings
☒	☐	For hierarchical and complex designs, identification of the appropriate level for tests and full reporting of outcomes
☒	☐	Estimates of effect sizes (e.g. Cohen's *d*, Pearson's *r*), indicating how they were calculated

Our web collection on statistics for biologists contains articles on many of the points above.

Software and code

Policy information about availability of computer code

Data collection	Las-X (Leica Microsystems, THUNDER Imager 3D Cell Culture and Leica SPE)<br>Zetasizer Nano ZS (Malvern)<br>TRIOS (TA Instruments)<br>StepOnePlus (Applied Biosystsems, v2.3)<br><br>G-Code to control the automated magnetic ink dispersion as well as the automated control over magnetic rod movement and electromagnetic field are included as 'Supplementary Methods' in the Supplementary Information.
Data analysis	ImageJ (NIH, v.2.1.0/1.53c)<br>CellProfiler (v4.2.5)<br>MATLAB R2019b<br>GraphPad Prism v.9.3.1

For manuscripts utilizing custom algorithms or software that are central to the research but not yet described in published literature, software must be made available to editors and reviewers. We strongly encourage code deposition in a community repository (e.g. GitHub). See the Nature Portfolio guidelines for submitting code & software for further information.

Data

Policy information about availability of data

All manuscripts must include a data availability statement. This statement should provide the following information, where applicable:

- Accession codes, unique identifiers, or web links for publicly available datasets
- A description of any restrictions on data availability
- For clinical datasets or third party data, please ensure that the statement adheres to our policy

All data associated with this manuscript has been deposited in a publicly available repository (the Stanford Digital Repository) under the persistent URL: https://purl.stanford.edu/sw198jy9339 and the DOI: https://doi.org/10.25740/sw198jy9339

Human research participants

Policy information about studies involving human research participants and Sex and Gender in Research.

Reporting on sex and gender	NA
Population characteristics	NA
Recruitment	NA
Ethics oversight	NA

Note that full information on the approval of the study protocol must also be provided in the manuscript.

Field-specific reporting

Please select the one below that is the best fit for your research. If you are not sure, read the appropriate sections before making your selection.

☒ Life sciences ☐ Behavioural & social sciences ☐ Ecological, evolutionary & environmental sciences

For a reference copy of the document with all sections, see nature.com/documents/nr-reporting-summary-flat.pdf

Life sciences study design

All studies must disclose on these points even when the disclosure is negative.

Sample size	Power analysis of sample size was not done prior to experimentation. Here, we describe the development of a new biofabrication approach and, as such, sample sizes were estimated empirically based on previous studies (Birey et. al., Nature 2017; Myura et al., Nature Biotechnology 2020; Ayan et al. Science Advances 2020; Daly et al. Nature Communnications 2021; Kim et al. Biofabrication 2022).
Data exclusions	No data was excluded from the analyses.
Replication	Data shown from representative experiments were repeated with similar results in at least 3 independent experiments, unless otherwise indicated by sample size (included in both the figure legend and a supplementary table with all the statistical comparisons). Unless otherwise noted in the figure legend, each distinct biological replicate is displayed as a data point superimposed on the associated plot.<br><br>The organoid printing approach was repeated with human induced plurpotent stem cell (hiPSC)-derived neural organoids from four distinct lines and two donors, two diffuse intrinsic pontine glioma (DIPG) lines, one pediatric glioblastoma (pGBM), one adult glioblastoma (aGBM), and one anaplastic oligodendroglioma (AO) cell line. Validation of organoid fusion into assemblopids across different cell types (hiPSC-derived neural organoids and all four primary tumor organoids), brain regions (dorsal and ventral forebrain), metastatic profiles (originating pons and metastasized forebrain), and donors demonstrates the reproducibility of this bioprinting method.
Randomization	All spheroids and organoids were randomly selected for specific assays.<br><br>All quantification of fluorescence images (e.g. Cleaved Caspase 3 and Histone H3K27M in DIPG organoids) involved analyzing whole samples, and no regional randomizations were applicable (i.e. the cell number for each marker was quantified across the entire organoid and normalized by every nucleus in said organoid).
Blinding	The investigators were not blinded to allocation during experiments and outcome measurements. Our data sets are based on objectively measurable data (fluorescent intensity, fluorescence area). Blinding does not affect these data values.

Reporting for specific materials, systems and methods

We require information from authors about some types of materials, experimental systems and methods used in many studies. Here, indicate whether each material, system or method listed is relevant to your study. If you are not sure if a list item applies to your research, read the appropriate section before selecting a response.

Materials & experimental systems

n/a	Involved in the study
☐	☒ Antibodies
☐	☒ Eukaryotic cell lines
☒	☐ Palaeontology and archaeology
☒	☐ Animals and other organisms
☒	☐ Clinical data
☒	☐ Dual use research of concern

Methods

n/a	Involved in the study
☒	☐ ChIP-seq
☒	☐ Flow cytometry
☒	☐ MRI-based neuroimaging

Antibodies

Antibodies used

Rabbit Anti-GFP (Invitrogen A-11122)
Rabbit Anti-Cleaved Caspase 3 (Cell Signaling 9661)
Rabbit Anti-Histone H3 (mutated K27M) (Abcam ab190631)
Rabbit Anti-PAX6 (Biolegend 901302)
Mouse Anti-NKX2.1 (Clone: 8G7G3/1) (Invitrogen ma5-13961)
Chicken Anti-TUBB3 (Aves Labs TUJ)
Chicken Anti-GFAP (Aves Labs GFAP)
Goat Anti-Rabbit, Alexa Fluor 488 (Invitrogen A-11034)
Goat Anti-Mouse, Alexa Fluor 594 (Invitrogen A-11020)
Goat Anti-Chicken, Alexa Fluor 633 (Invitrogen A-21103)
4',6-diamidino-2-phenylindole (DAPI) (Invitrogen D1306)

Validation

Validation Validation and references on manufacturer's website.

Anti-GFP (1:200) - 647 publications; https://www.thermofisher.com/order/genome-database/dataSheetPdf?producttype=antibody&productsubtype=antibody_primary&productId=A-11122&version=278

Anti-Cleaved Caspase 3 (1:400) - 13496 publications; https://media.cellsignal.com/coa/9661/47/9661-lot-47-coa.pdf

Anti-Histone H3 (mutated K27M) (1:400) - 4 publications; https://www.abcam.com/histone-h3-mutated-k27m-antibody-epr18340-chip-grade-ab190631.html

Rabbit Anti-PAX6 (Biolegend 901302) - 308 publications; https://www.biolegend.com/nl-be/products/purified-anti-pax-6-antibody-11511

Mouse Anti-NKX2.1 (Invitrogen ma5-13961) - 68 publications; https://www.thermofisher.com/order/genome-database/dataSheetPdf?producttype=antibody&productsubtype=antibody_primary&productId=MA5-13961&version=302

Chicken Anti-TUBB3 (Aves Labs TUJ) - 38 publications; https://cdn.shopify.com/s/files/1/0512/5793/4009/files/TUJ_datasheet.pdf

Chicken Anti-GFAP (Aves Labs GFAP) - 55 publications; https://cdn.shopify.com/s/files/1/0512/5793/4009/files/GFAP_datasheet.pdf

Alexa Fluor 488 (1:500) - 5626 publications; https://www.thermofisher.com/order/genome-database/dataSheetPdf?producttype=antibody&productsubtype=antibody_secondary&productId=A-11034&version=278

Alexa Fluor 594 (Invitrogen A-11020) - 225 publications; https://www.thermofisher.com/order/genome-database/dataSheetPdf?producttype=antibody&productsubtype=antibody_secondary&productId=A-11020&version=302

Alexa Fluor 633 (Invitrogen A-21103) - 85 publications; https://www.thermofisher.com/order/genome-database/dataSheetPdf?producttype=antibody&productsubtype=antibody_secondary&productId=A-21103&version=302

DAPI (1:2000) - https://www.thermofisher.com/document-connect/document-connect.html?url=https://assets.thermofisher.com/TFS-Assets%2FBID%2Fcertificate%2FCertificates-of-Analysis%2FD1306%20Lot%202500455%20CofA.pdf

Eukaryotic cell lines

Policy information about cell lines and Sex and Gender in Research

Cell line source(s)

Human mesenchymal stromal cells (Lonza PT-2501)
Human umbilical vein endothelial cells (Lonza C2519A)

Human induced pluripotent stem cells (hiPSCs):
5(Male, SCRO #267; Obtained from Prof. Theo Palmer at Stanford University)
511.3 (Male, SCRO #267; Obtained from Prof. Theo Palmer at Stanford University)
SUN004.1.9 (Male, IRB #35445; Obtained from Prof. Kyle Loh at Stanford University)
SUN004.2 (Male, IRB #35445; Obtained from Prof. Kyle Loh at Stanford University)

Diffuse intrinsic pontine glioma (DIPG)-XIII (Female, Obtained from Prof. Michelle Monje at Stanford University)
Pediatric glioblastoma (pcGBM-2) (Male, Obtained from Prof. Michelle Monje at Stanford University)
Adult glioblastoma (GBM-81) (Male, Obtained from Prof. Michelle Monje at Stanford University)
Anaplastic oligodendroglioma (SU-AO-3) (Male, Obtained from Prof. Michelle Monje at Stanford University)

Authentication

Commercial cell lines were authenticated by the vendor.

All other lines were thoroughly characterized and authenticated in previously published manuscripts (Roth et al. eLife 2020; Ang et al. Cell 2022; Grasso et al. Nature Medicine 2015, Nagaraja et al. Cancer Cell 2017, Lin et al. Science Translational Medicine 2019).

Mycoplasma contamination

All cell lines were routinely tested for mycoplasma contamination and tested negative.

Commonly misidentified lines
(See ICLAC register)

No commonly misidentified lines were used.
